# Is there a difference in the effect between the ACEI and ARB on COVID‐19?

**DOI:** 10.1002/clc.23474

**Published:** 2020-11-18

**Authors:** Jie Chen, Kaibo Mei, Chao Deng

**Affiliations:** ^1^ Cardiology Department The Third Affiliated Hospital of Nanchang University Nanchang China; ^2^ Anesthesiology Department The People's Hospital of Shangrao Shaorao China; ^3^ Cardiology Department Affiliated Hospital of Jiangxi University of traditional Chinese Medicine Nanchang China

To the Editor

We read with great interest the article by Liu et al. about the impact of angiotensin‐converting enzyme inhibitors and angiotensin II receptor blockers (ACEI/ARB) on coronavirus disease‐19 (COVID‐19).^1^ The authors found that ACEI/ARB therapy was not associated with increased risk of COVID‐19 and did not have a worse prognosis. Yet, we have some concerns. Firstly, the potential difference between ACEI/ARB was not examined and discussed thoroughly. As we all know, severe acute respiratory syndrome coronavirus 2 (SARS‐CoV‐2) uses angiotensin‐converting enzyme 2(ACE2) for cell entry by binding with its spike protein to ACE2.^2^ However, treatment with ACEIs and ARBs might have a differential impact on several components of the renin‐angiotensin system (RAS). A review showed that, in animals' experiments, a more consistent up‐regulation of ACE2 was observed for ARBs, while the modulation of ACE2 by ACEIs was more variable.^3^ Furthermore, in human studies, a prospective cohort study (n = 617) showed that urinary ACE2 level was higher in the ARB‐treated for 1‐year group, but not the ACEI treatment groups, than in the no‐medication treatment control individuals.^4^ Consistently, a multicenter cohort consisting of 8910 patients hospitalized with COVID‐19 showed ACE inhibitor use is associated with a lower risk of hospital death (odds ratio: 0.33), however, an increased risk for ARB use(odds ratio: 1.23).^5^ Therefore, a subgroup analysis or more discussion in exploring the potential difference in the effect on the COVID‐19 between ACEI and ARB might be necessary.

Another critical issue is whether there is a positive exposure‐effect relationship between the numbers of ACE2 and the risk of COVID‐19. The most recent evidence derives from the COVID‐19 and IBD (inflammatory bowel disease). It has been shown that there is a higher ACE2 protein expression in terminal ileum and colon and soluble ACE2 in patients with IBD compared with controls.^6^ However, up to date, there is currently no evidence for increased risk or aggravated outcomes in patients with IBD in the context of COVID‐19.^7^

Finally, the effect of RAS on immune response was mentioned in the title, however, it is not explained in the introduction and discussion. Inflammation certainly plays a pivotal role in the COVID‐19. RAS has been shown to inhibit pro‐inflammatory immune responses (eg, interleukin‐6, tumor necrosis factor‐alpha) and to alleviate the acute lung injury caused by viruses, such as SARS.^8^ More recently, tocilizumab‐interleukin‐6 antibody, is considered for the severe COVID‐19 treatment and a prospective phase III trial has been initiated (www.clinicaltrialsarena.com/new/roche-actemra-covid-19-trial). The results of this trial were helpful to clarify its mechanism of ACEI/ARB on immune response in COVID‐19.

## CONFLICT OF INTEREST

The authors declare no potential conflict of interests.

## Data Availability

Data sharing is not applicable to this article as no new data were created or analyzed in this study.
